# Blood Platelets in Infection: The Multiple Roles of the Platelet Signalling Machinery

**DOI:** 10.3390/ijms24087462

**Published:** 2023-04-18

**Authors:** Silvia M. G. Trivigno, Gianni Francesco Guidetti, Silvia Stella Barbieri, Marta Zarà

**Affiliations:** 1 Department of Biology and Biotechnology, University of Pavia, 27100 Pavia, Italy; 2 University School for Advanced Studies, IUSS, 27100 Pavia, Italy; 3 Unit of Heart-Brain Axis: Cellular and Molecular Mechanisms, Centro Cardiologico Monzino IRCCS, 20138 Milano, Italy; marta.zara@ccfm.it

**Keywords:** platelets, infection, inflammation

## Abstract

Platelets are classically recognized for their important role in hemostasis and thrombosis but they are also involved in many other physiological and pathophysiological processes, including infection. Platelets are among the first cells recruited to sites of inflammation and infection and they exert their antimicrobial response actively cooperating with the immune system. This review aims to summarize the current knowledge on platelet receptor interaction with different types of pathogens and the consequent modulations of innate and adaptive immune responses.

## 1. Introduction

Infectious diseases are an unmet human health challenge and remain a major cause of morbidity and mortality worldwide, especially in resource-limited countries.

The entry of a pathogen into the bloodstream could represent a lethal threat to the organism, and therefore containment of the infection is attempted by all available means. In vertebrates, two types of immunity protect the host from infection: innate and adaptive immunity. The innate immune system is genetically programmed to recognize invariant features of invading microbial pathogens, whereas the adaptive immune system employs antigen-specific receptors.

Platelets are classically known as essential mediators of haemostasis and thrombosis, but they are also involved in many other physiological and pathological processes, including angiogenesis, atherosclerosis, tumour progression, and infection. It is now clear that platelets are among the first cells recruited to sites of inflammation and infection, and they play a fundamental role in both processes. The immune effects of platelets can be observed both locally at the site of platelet activation and deposition and systemically at sites distant from the platelet activation itself.

In this review, we will discuss the important role of platelets in infections, focusing on the specific platelet receptors involved and on the shedding of platelet surface proteins. As in many other fields, the COVID-19 pandemic strongly boosted the research on platelets in the SARS-CoV-2 infection. However, in this review, we did not focus on this specific aspect since it has been widely described in several recent papers [1,2].

## 2. Platelets in Infection

**Figure 1:**
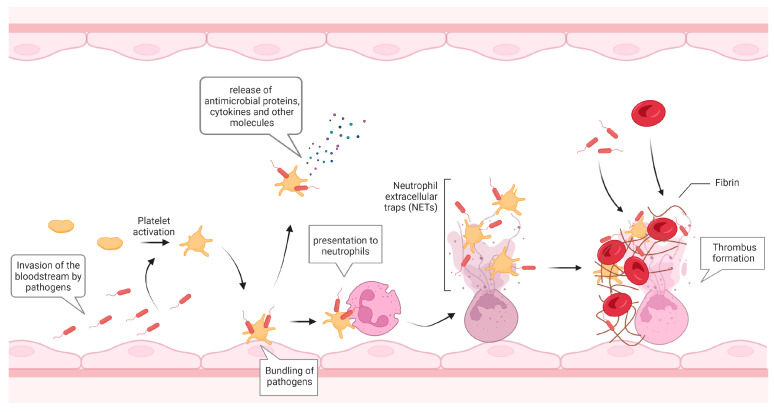
Schematic representation of platelet response to pathogen infection. Created with BioRender.com (Licensing number HN254B4IYS).

Platelets are among the first cells recruited to sites of inflammation and infection and play an essential role in initiating intravascular immune responses through complex cooperation with white blood cells and vascular endothelial cells. The interplay between platelets and immune cells is also flanked by coagulation and complement systems, all of which form an intertwined process linking inflammation and thrombosis (Figure 1).

Physiologically, the inflammation-dependent activation of the coagulation system is part of the host response to pathogens aimed at limiting their systemic spread in the bloodstream. This complex set of responses, involving platelets, immunity, and coagulation, has been defined with the general term of immunothrombosis. Moreover, inflammation, which has been classically considered an entirely separated unit, is a common response occurring upon wounds, tissue damage, and thrombotic events. It is now clear that thrombosis and inflammation should be investigated as a single entity and inflammatory markers can be exploited to better understand the physiopathology of thrombosis [3].

Although platelets actively participate in both innate and adaptive immunity, they are considered fundamental players in innate immunity, expressing several different pathogen recognition receptors (PRRs). Through these receptors, platelets act as vascular sentinels that interact with pathogens and exert antimicrobial activity by mediating both direct and indirect effects, as summarized in Table 1.

Platelets exploit actomyosin-dependent forces to migrate and scan the surrounding microenvironment. After recognizing and binding the pathogens, platelets shuttle them to antigen-presenting cells and boost the activity of professional phagocytes [7,8,25,26]. However, migrating platelets also directly engulf bacteria and viruses, as demonstrated by *Staphylococcus aureus* and human immunodeficiency virus (HIV) [6,10]. They actively translocate the pathogens to the open canalicular system (OCS) invaginations, thus effectively preventing their blood-borne dissemination. Microbe collection by migrating platelets can exert additional functional responses. For example, HIV virions, after being trapped in the OCS, come into contact with Platelet Factor 4 (PF4, CXCL4), which inhibits virus replication [27].

During infection, receptor-mediated platelet activation is also accompanied by the secretion of α-granules and dense granules that store molecules with antimicrobial activity and/or immunomodulatory effect (Table 2). 

The most abundant antimicrobial protein contained in α-granules is the abovementioned PF4, whose activity is well characterized for malaria parasites [29,44] but also bacteria and viruses. Platelet α-granules also contain α- and β-defensins that display antibacterial activity against *Escherichia coli* [45] and *Staphylococcus aureus* [10], respectively. In addition to direct antimicrobial activity, other molecules stored in α-granules (mainly CD40L and TGFβ) have the ability to shape the immune systems. Both soluble and membrane-bound CD40L mediate immunomodulatory activities by binding to the CD40 expressed in the immune cells. Soluble CD40L released by activated platelets stimulates the dendritic cells, resulting in increased phagocytosis and the intracellular killing of bacteria [32]. The CD40L expressed on the platelet surface also plays a key role in supporting antibody isotype switching (e.g., from IgM to IgG) and enhancing CD8+ T-cell function [20,21]. Moreover, the binding of platelet-derived CD40L to CD40 upregulates the expression of the adhesion molecules and secretion of pro-inflammatory cytokines by the endothelial cells, thus promoting the recruitment of leukocytes at the sites of infection [23,46,47]. Platelets also contribute significantly to increasing the circulating levels of TGF-β, and various mechanisms have been proposed for its immunoregulatory functions. Platelet-derived TGF-β regulates the differentiation of CD4+ T cells into regulatory T cells [18,48], which are immunosuppressive and help maintain tolerance toward self-antigens. The importance of the platelet TGF-β in the differentiation of regulatory T cells was clearly demonstrated by the observation that regulatory T-cell concentration and function are impaired in thrombocytopenic disorders [49,50]. Stromal cell-derived factor-1 (SDF-1, CXCL12) is another potent, platelet-derived, chemoattractant and modulator of immune responses mediated by monocytes and lymphocytes [38], dendritic cells [39], and endothelial cells [40,51]. PF4, itself released from platelet α-granules, is involved in the recruitment and activation of leukocytes. Similarly, the platelet-derived growth factor (PDGF) attracts monocytes to the site of the vascular injury [52], stimulates eosinophils to generate superoxide anions [34], and could limit these pro-inflammatory events through the autocrine feedback inhibition of platelet aggregation.

Finally, the release of α-granules results in the exposure of P-selectin, which is responsible for the platelet–leukocyte interaction, a common feature in infectious diseases [53,54,55,56]. The formation of platelet–leukocyte aggregates leads to the stimulation of leukocyte signalling pathways culminating in the release of bactericidal/pro-inflammatory molecules, thromboinflammation, and/or the phagocytosis of the pathogen [55,57,58].

Molecules known to play a central role in platelet activation in the frame of haemostasis and thrombosis may also act as inflammatory/immune modulators. The von Willebrand factor (VWF) promotes inflammation, increasing the extravasation of neutrophils [35,36,37]. Similarly, ADP, released from platelet-dense granules, increases antigen endocytosis and processing [41] in dendritic cells through binding to its receptor, P2Y_12_. Recently, a number of immunoregulatory functions have also been ascribed to serotonin, another important molecule released from platelet-dense granules during cell activation. Almost all immune cells express serotonin receptors, and, therefore, serotonin exerts functions in innate, as well as adaptive, immunity; for example, by modulating cytokine release from monocytes/macrophages, neutrophil recruitment, and T-cell activation [42,43].

The importance of platelets in infection is further supported by the observation that thrombocytopenia (i.e., a low platelet count) represents a hallmark of poor prognoses in many infectious diseases [59,60]. Patients with sepsis associated with thrombocytopenia usually have a worse prognosis and increased mortality if compared with patients with normal platelet counts [61]. This link between platelet number and clinical outcome has been confirmed in several mouse models, and platelet-depleted mice typically displayed increased pathogen dissemination and increased mortality compared to mice with normal platelet counts [62,63,64].

## 3. Platelet Receptors in the Response to Infection

**Figure 2:**
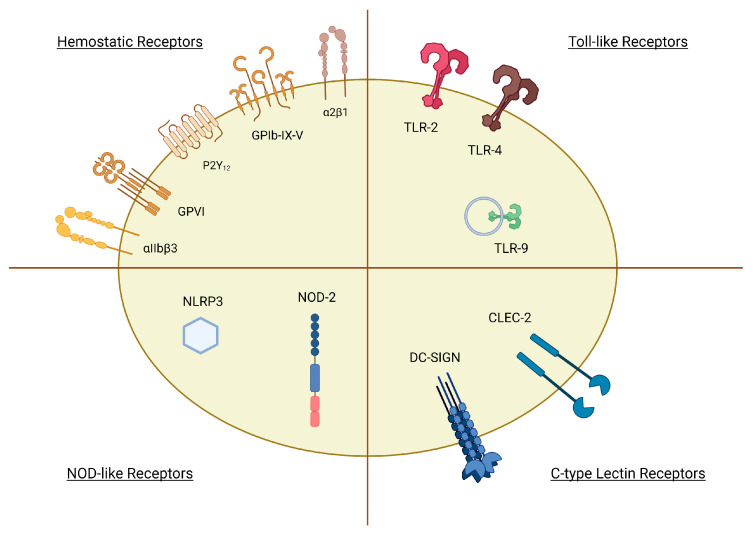
Platelets sense pathogens and host damage through recognition of PAMPs or DAMPs using receptors. Toll-like receptors (TLRs) include surface receptors TLR2 and 4 and endosomal TLR9. NOD-like receptors (NLRs) include NLRP3 and NOD-2. C-type lectin receptors (CLRs) include DC-SIGN and CLEC-2. Haemostatic platelet receptors: GPIb, GPVI, integrin αIIbβ3, P2Y_12_, and integrin α2β1. Created with BioRender.com (Licensing number MY2549P1ZK).

The ability of platelets to recognize viral, parasitic, and bacterial infections and to stimulate specific responses is mediated both by PRRs, which are specialized for pathogen recognition during infection, and by receptors primarily involved in the haemostatic/thrombotic response (haemostatic receptors) (Figure 2) [65].

These receptors initiate complex and only partially known signal transduction pathways that cooperate to support the functional role of platelets during infection. Table 3 summarizes the known ligands of platelet receptors involved in the response to infection.

### 3.1. Pattern Recognition Receptors (PRRs)

PRRs were originally discovered in innate immune cells and are involved in the primary defence against infectious diseases. PRRs recognize two distinct groups of molecular patterns: damage-associated molecular patterns (DAMPs) and pathogen-associated molecular patterns (PAMPs). DAMPs are classically defined as endogenous danger signals, and they are released by host cells under stress conditions, such as tissue damage. Conversely, PAMPs are derived directly from pathogens and thus represent exogenous signals for the host. Bacterial membrane/wall or viral capsid components and nucleic acids, particularly the unmethylated CpG motifs, are some examples of PAMPs. Three classes of PRRs are known to be expressed by platelets: toll-like receptors (TLRs), C-type lectin receptors (CLRs), and NOD-like receptors (NLRs), whereas RIG-I-like receptors (RLRs) have not yet been found in platelets [97].

#### 3.1.1. TLRs

The most abundantly expressed receptors on platelets are TLR2-4-9, whereas TLR1-3-5-6-8 receptors are lower expressed. Due to the low number of studies examining TLRs 7 and 10, it is difficult to determine the extent of their expression on/within platelets. TLRs can recognize both PAMPs and DAMPs and mediate several responses in platelets, such as aggregation, their interaction with leukocytes, and the release of inflammatory mediators. The role of platelet TLRs in infection is not discussed in detail here since topic-specific reviews have recently been published [98,99,100].

#### 3.1.2. CLRs

CLRs are specialized in the recognition of glycans through their conserved carbohydrate-binding domains. Two members of the CLR family, namely C-type lectin-like receptor 2 (CLEC-2) and dendritic cell-specific ICAM-3-grabbing non-integrin 1 (DC-SIGN), are abundantly expressed in platelets.

CLEC-2 (also known as CLEC1B) is a type II transmembrane receptor, and it has been identified on platelets as the major receptor for the platelet-activating snake venom, aggretin. It is involved in the stabilization of clots, and it plays an important role in inflammation by binding podoplanin on the macrophages and by supporting direct pathogen recognition and interaction [101].

CLEC-2 has been shown to mediate Dengue Virus (DV)-induced platelet activation by stimulating the secretion of platelet α- and dense-granules and the release of extracellular vesicles (EVs). EVs released upon DV infection can interact with CLEC5A and TLR2 receptors on the surface of neutrophils and macrophages [67], promoting their recruitment to the site of infection, the NETs’ formation, and the release of pro-inflammatory cytokines [102].

In HIV infection, CLEC-2 directly mediates viral capture and internalization in closed endosomal structures, a process that occurs preferentially in activated platelets [6,66]. After this internalization, the chemokine, PF4, interacts with the major HIV envelope glycoprotein (GP120) and inhibits viral replication.

CLEC-2 has also been described as the main receptor involved in platelet responsiveness to bacterial unmethylated CpG-rich DNA. Synthetic CpG-containing oligodeoxynucleotides (CpG-ODNs) are able to mimic the effect of viral/microbial DNA and to elicit a strong immune response, including the secretion of cytokines and chemokines and the activation of B and T cells, monocytes, NK cells, and antigen-presenting cells (APCs). Platelets are able to interact with and internalize CpG ODN in a process that likely depends on multiple receptors, including CLEC-2, GPVI, TLR-9, and P2Y_12_. Through CLEC-2, CpG-ODNs induce platelet activation by stimulating Src- and Syk-dependent pathways, ultimately promoting PLCγ2 activation [68]. This process also leads to P-selectin expression, platelet aggregation, and the formation of platelet–neutrophil and platelet-monocyte aggregates [81].

DC-SIGN (also known as CD209) is a type II transmembrane glycoprotein that recognizes mannose-containing pathogen-associated carbohydrates. It is expressed mainly on the surface of macrophages and dendritic cells, but it has also been detected in platelets. Similarly to CLEC-2, DC-SIGN is involved in the interaction/engulfment of HIV and DV and thus their inhibition through PF4 activity. It has been observed that the combination of pharmacological inhibitors targeting these two lectins greatly reduces the binding of virions to platelets and decreases their internalization. Nevertheless, the actual role of platelets in HIV and DV infection remains controversial. As mentioned above, platelets produce and release molecules that negatively affect the virus’s lifespan, including PF4. On the other hand, they serve as shelters for virions during immune cell attacks and aid in the transport of the virus in the bloodstream. In fact, the interaction between platelets and HIV facilitates viral spread through the bloodstream and participates in the development of thrombocytopenia, which is frequently observed in HIV/AIDS patients. Moreover, it was recently discovered that the DV is able to enter the platelet cytosol in a DC-SIGN-dependent manner and undergo decapsidation, releasing the ssRNA content. The viral genome is then replicated, new nucleocapsids are assembled in the platelet Golgi apparatus, and finally, viral particles are released into the bloodstream [69].

#### 3.1.3. NLRs

Platelets express two cytoplasmic PRRs belonging to the NLR family: the NACHT, LRR, and PYD domains-containing protein 3 (NLRP3) and the nucleotide-binding oligomerization domain-containing protein 2 (NOD2) [103].

NLRP3 operates as a cytoplasmic sensor to activate the inflammasome, and it recognizes tissue damage signals such as reactive oxygen species (ROS) generated upon the cell–PAMP interaction. NLRP3 is abundant in macrophages but is also constitutively expressed in platelets, where it supports the synthesis of pro-inflammatory cytokines. Specifically, the infection of platelets by DV induces ROS production by the mitochondria in a RIP1/RIP3-dependent manner that activates NLRP3. Active NLRP3 recruits the apoptosis-associated speck-like protein (ASC), which in turn activates caspase-1 and leads to the formation of the inflammasome that controls the secretion of the pro-inflammatory cytokine IL-1β. In platelets from DV-infected patients, active NLRP3-inflammasomes cleave the full-length pro-IL-1β into the mature IL-1β, which accumulates in the platelets and is then sorted into EVs. These platelet-derived EVs eventually interact with endothelial cells and increase vascular permeability, contributing to the vasculopathy of Dengue [104]. In addition, IL-1β can bind to the IL-1 receptor (IL-1R) exposed on the platelet surface, leading to the phosphorylation of c-Src and Syk and regulation of platelet spreading and clot retraction via integrin αIIbβ3 outside-in signalling [105]. Intriguingly, selective NLRP3 inhibitors have been shown to ameliorate platelet defects associated with DV, suggesting that the NLRP3 inflammasome may be a novel target for the treatment of Dengue-associated thrombocytopenia [76].

A recent study demonstrated the co-localization of NLRP3 and ASC in platelets upon LPS-induced stimulation and in platelets isolated from cecal ligation puncture (CLP)-induced septic rats. In the same experimental model, increased caspase-1 activity and IL-1β secretion were also observed, which were associated with impaired endothelial permeability and multiple organ damage [106]. The same research group later showed that treatment of CLP mice with a specific NLRP3 inflammasome inhibitor (MCC950) significantly attenuated platelet activation and multi-organ damage induced by sepsis [107]. Another inhibitor of NLRP3 inflammasome activation identified in platelets is Ibrutinib, an inhibitor of Bruton’s tyrosine kinase (BTK), but its potential efficacy in sepsis has not yet been established [108].

The increased expression of NLRP3 and cleavage of IL-1β were observed in platelets from patients infected with chikungunya virus, suggesting that the platelet–inflammasome engagement may be a relatively common response to viral infection [109]. 

NOD2 is a cytoplasmic protein abundantly expressed in monocytes and dendritic cells, and it has recently been detected in platelets. During infection, Gram-positive and Gram-negative bacteria can release several immunomodulatory components in the bloodstream, including the cell wall fragment, muramyl dipeptide (MDP). Circulating MDPs can be transported by endosome recycling into the cellular cytosol, where it can interact with its receptor, NOD2 [110]. The selective stimulation of NODs by MDPs potentiates platelet activation and aggregation, suggesting that this receptor may contribute to platelet responsiveness to infection. Moreover, the MDP-mediated activation of NOD2 promoted IL-1β accumulation in human and mouse platelets in a caspase-1-dependent manner [77]. Moreover, platelets from septic patients or mice with CLP-induced sepsis show a stronger expression of P2Y_12_ receptors promoted by a NOD2-dependent pathway. Indeed, P2Y_12_ overexpression was attenuated in septic NOD2-deficient mice [111]. 

### 3.2. Haemostatic Receptors

Platelet membrane receptors are essential components of the molecular machinery that supports platelet activation in haemostasis and thrombosis. However, some key haemostatic receptors also play important roles in pathogen recognition and platelet activation, supporting immunothrombosis and other defenceresponses.

Glycoprotein VI (GPVI) is selectively expressed by platelets and their progenitor cells and, together with integrin α2β1, is the major platelet collagen receptor. On the plasma membrane, GPVI is physically associated with the γ-chain of the FcR immunoglobulin receptor (FcR-γ chain), which carries the immunoreceptor tyrosine-based activation motif (ITAM) essential for collagen-induced signal transduction. In addition to its well-known role in haemostasis/thrombosis, GPVI is also involved in the response to viral and bacterial infections.

Human GPVI has been identified as an interactor of hepatitis C virus (HCV) through the extracellular immunoglobulin (Ig)-like domains and plays a relevant role in viral transport and persistence [78]. GPVI has also been implicated in infection with Gram-negative bacteria, including *Klebsiella pneumoniae*, and Gram-positive bacteria, such as *Staphylococcus aureus*. In sepsis caused by *Klebsiella pneumonia*, platelet GPVI is involved in host defence by supporting platelet recruitment and activation to the site of infection, thereby affecting the formation of platelet–leukocyte aggregates, leukocyte activation, and bacteria phagocytosis [79]. Staphylococcal superantigen-like protein 5 (SSL5) is an exotoxin secreted by *Staphylococcus aureus* that induces platelet activation, adhesion, and aggregation through interaction with GPVI, as well as GPIbα and integrin αIIbβ3 [80,82]. Platelet activation mediated by SSL5 has a detrimental effect on prognosis, as it can lead to disseminated intravascular coagulation (DIC) associated with multiple organ failure and thrombocytopenia. The importance of GPVI in infection has recently been confirmed in septic patients. Platelets from patients diagnosed with sepsis were found to be hyporeactive, and the GPVI signalling cascade was severely impaired. The mechanisms underlying this phenomenon remain to be explored, but the shedding of the ectodomain of the GPVI has been proposed as one of the possible causes [112].

Glycoprotein Ib (GPIb) (also known as CD42) is a component of the GPIb-V-IX complex and binds vWF, allowing platelet rolling and adhesion to the injury site under high shear stress. GPIb has been shown to interact with several types of bacterial PAMPs, including the *Streptococcus sanguis* platelet adhesin called serine-rich protein A (SrpA) [83], the surface proteins of *Streptococcus gordonii* GspB and Hsa [84], and also the Protein A (Spa) expressed on the surface of *Staphylococcus aureus*. In the latter case, recognition of Spa by GPIb does not occur directly but is mediated by soluble vWF. This mechanism is a typical example of how soluble molecules can act as mediators of binding between host cells and pathogens [85]. In this context, vWF, with the support of IgG, also mediates the interaction of GPIb with *Helicobacter pylori* [86].

Integrin αIIbβ3 (also known as GPIIbIIIa or CD41/CD61) is the most abundant platelet membrane receptor and supports several key responses by binding RGD containing proteins such as fibrinogen and fibronectin. Integrin αIIbβ3 also mediates interaction with pathogens, including Hantavirus [87,113] and Adenovirus [88], and different bacterial strains. The fibrinogen-binding protein serine–aspartate repeat protein G (SdrG), also known as Fbe, from *Staphylococcus epidermis* is a critical interactor of integrin αIIbβ3. It is present in most clinical strains and causes platelet aggregation via both direct and indirect interaction. A direct interaction occurs between the B domain of SdrG and integrin αIIbβ3, while an indirect interaction involves the binding of fibrinogen and the IgG receptor, FcγRIIa [89].

Another indirect interaction between bacteria and platelets is mediated by fibrinogen and clumping factors in *Staphylococcus aureus*. Like the Sdr proteins, clumping factors A (ClfA) and B (ClfB) contain serine and aspartic acid dipeptide repeats (SD repeats) that link the transmembrane region to the ligand binding domain. The latter binds fibrinogen and induces platelet aggregation in an αIIbβ3-dependent manner but also in an IgG-dependent manner [93]. In addition, the fibrinogen/fibronectin-binding proteins FnBPA and FnBPB were involved in *Staphylococcus aureus*-induced platelet activation. It has been shown that the binding of FnBP to αIIbβ3 via fibrinogen and fibronectin bridges in the presence of FnBP-specific antibodies is required for full platelet activation [92]. Other important binding partners for integrin αIIbβ3 that support adhesion to pathogens and platelet aggregation are the platelet adherence protein A (PadA) of *S. gordonii* [90] and surface determinant (Isd) proteins of *Staphylococcus aureus* [91].

The human platelet FcγRIIa receptor (also called CD32a) is a low-affinity receptor for the constant region of IgG, thus mediating the binding of immune complexes and IgG-opsonized cells. As described above, platelet interaction with different pathogens, including *Staphylococcus aureus* and *Staphylococcus epidermis*, and subsequent activation can be supported by IgG. The signalling through FcγRIIa is based on a tyrosine kinase cascade and results in Ca^2+^ mobilization that enhances platelet activation [95].

Interestingly, virus-mediated platelet responses are also dependent on FcγRIIa. Immunocomplexes of the influenza A (H1N1) virus with cross-reactive IgG activate platelets through FcγRIIA, increasing platelet degranulation and microparticle shedding [96].

Platelets express additional Fc receptors, including FcεR and FcαRI, which enable the scavenging of circulating IgE- and IgA-containing immune complexes, respectively. Platelet FcεR (CD23) is known to play a role in allergic reactions [114], but it was also proposed as a possible mediator of platelet response to *Schistosoma mansoni* parasite infection in rats [115]. FcαRI (CD89) mediates platelet activation through Src family kinases and induces the release of tissue factor (TF) and IL-1β, suggesting a possible involvement for human platelet FcαRI and serum IgA in thrombosis and inflammation [116].

## 4. Shedding of Platelet Surface Proteins in Infection

As described, membrane receptors are essential for the recognition of infectious agents. Another mechanism involving platelet membrane proteins in the context of infection/inflammation is proteolytic shedding. Platelet transmembrane protein shedding occurs when the extracellular portion of the protein is cleaved by specific enzymes commonly referred to as sheddases, releasing the soluble fragment into the surrounding environment. This process may act as a control mechanism to reduce the receptor density on the platelet surface, limiting their responsiveness and thrombus formation. Moreover, the soluble ectodomain can mediate functional responses in several target cells.

Only a limited proportion (approximately 10%) of platelet membrane proteins are thought to be subject to protein shedding. Using a proteomic approach, 69 platelet membrane proteins have been identified as candidates for shedding [117], but activation-induced shedding has been experimentally demonstrated for a few of them [118]. Since platelet activation also occurs during inflammation triggered by pathogens, the soluble fragments shed from the platelet surface may represent novel potential biomarkers for infection [119,120]. The major platelet proteins whose shedding has gained interest in the context of infection are described below.

### 4.1. sCD40L

The most studied platelet soluble fragment in infection and inflammation is the sCD40L (also known as CD154; MW 16 kDa). sCD40L is the soluble form of the trimeric transmembrane protein CD40L, which is critical for cell signalling in innate and adaptive immunity. In resting platelets, CD40L is stored in α-granules and, upon activation, it is rapidly exposed on the platelet surface [121], where it can act by binding to CD40 or the integrins on platelets and immune cells. Membrane CD40L can also be cleaved by the metalloproteinases MMP-2 and MMP-9, resulting in the release of sCD40L, which has cytokine-like activity. Interestingly, although CD40L is mainly expressed in immune cells, more than 95% of circulating sCD40L in blood originates from platelets [122]. The normal range of sCD40L in the serum of healthy individuals is estimated to be 0.79 to 4.7 ng/mL [123,124]. Elevated levels of sCD40L have been found in several infectious diseases, including *Pseudomonas aeruginosa* infection in cystic fibrosis patients [125], visceral leishmaniasis caused by *Leishmania infantum* [126], meningococcal sepsis [127], DENV-2 [45], HIV [128], and periodontopathogens infections [129].

In particular, platelet-derived sCD40L plays a fundamental role in the pathophysiology of abdominal sepsis. In a mouse model of sepsis induced by CLP, Rahman and co-workers showed that abdominal sepsis was associated with increased plasma levels of sCD40L and a concomitant reduced expression of CD40L on the platelet surface. In the same study, platelet depletion was shown to strongly decrease the plasma levels of sCD40L, confirming its possible platelet origin [130]. CD40L shedding and the consequent regulation of sCD40L plasma levels under septic conditions are strongly dependent on Rac1 signalling. In activated platelets, Rac1 controls the surface mobilization of CD40L, whereas, in neutrophils, Rac1 regulates MMP-9 secretion, which in turn promotes the proteolytic cleavage of platelet CD40L [131]. Once released into the extracellular environment, platelet-derived sCD40L can act as a ligand of Mac-1 (integrin αMβ2) expressed on the surface of neutrophils and promoting their activation and subsequent adhesion and migration but also the formation of platelet–neutrophil aggregates [132].

sCD40L levels are affected by the auto-amplification loop of platelet activation, in which sCD40L interacts with platelet CD40 and integrin αIIbβ3, resulting in further platelet activation and an increased release of sCD40L [124]. In this context, targeting platelet CD40L’s metabolism has been considered an alternative therapeutic strategy for a wide range of inflammatory disorders. For instance, the use of antiplatelet agents that can limit the release of sCD40L from platelets while leaving surface-expressed co-stimulatory CD40L on the T cells unchanged, such as integrin αIIbβ3 antagonists [133] or clopidogrel [134], has been proposed for the treatment of HIV-associated neuroinflammation without side effects on the humoral immune response [135].

### 4.2. sTLT-1

TREM-like transcript 1 (TLT-1) is an immunoreceptor tyrosine-based inhibition motif (ITIM)-containing receptor packaged into α-granules of resting platelets. Activation-induced platelet degranulation supports the exposure of TLT-1 on the plasma membrane, where it operates as a fibrinogen receptor. TLT-1 actively contributes to intracellular platelet signalling by interacting with cytoplasmic ERM proteins and modulating actin cytoskeleton polymerization [136].

Upon platelet activation, TLT-1 can be proteolytically cleaved and released as a soluble fragment (sTLT-1, 17 kDa) into the extracellular environment [137], and it is the fourth most abundantly released molecule in the supernatant of activated platelets [117]. sTLT-1 has not been found in the plasma of healthy individuals and mice, whereas it has been detected under conditions associated with platelet activation, including inflammation. High levels of sTLT-1 were found in the plasma of septic patients and mice injected with LPS, suggesting that sTLT-1 may be a meaningful marker of septicemia. It has also been shown that the addition of a recombinant sTLT-1 to ADP- or U46619-activated platelets enhanced platelet aggregation in a dose-dependent manner. The same study demonstrated an association between the plasma levels of sTLT-1 and the development of DIC, a condition frequently related to sepsis, suggesting sTLT-1 as a prognostic indicator of adverse outcomes [136,138]. sTLT-1 also displayed anti-inflammatory activity by binding TREM-1 expressed on neutrophils and monocytes and inhibiting its receptor activity. In several mouse models of septic shock, blocking TREM-1 signalling reduced organ damage and animal mortality. Therefore, the development of a synthetic mimetic of platelet-derived sTLT-1 may be a potential new approach to control the inflammation associated with sepsis [117].

### 4.3. sP-Selectin

Soluble P-selectin (sP-selectin, sCD62P, 93 kDa) has been detected in the plasma of healthy humans and mice at a concentration of 15 to 100 ng/mL, and its levels are increased in cardiovascular diseases such as atherosclerosis, hypertension, and myocardial infarction [139]. Like TLT-1, P-selectin (also known as CD62P) is stored in the α-granules of platelets and exposed on the cell membrane surface following platelet activation. However, P-selectin is also expressed by endothelial cells. Platelet P-selectin plays a key role in the formation of platelet–leukocyte aggregates by interacting with P-selectin glycoprotein ligand-1 (PSGL-1) expressed by leukocytes. This interaction is crucial for leukocyte recruitment and their rolling on activated platelets, supporting the inflammation process. It has been proposed that the interaction between platelets and leukocytes is involved in the shedding of P-selectin since levels of circulating sP-selectin are significantly reduced in PSGL-1-deficient mice [140]. Based on the increased shedding of the sP-selectin observed under inflammatory conditions, it has been proposed as a biomarker for infection. Particularly elevated levels of sP-selectin have been detected in patients with abdominal sepsis or skin infections [141]. Periodontopathogens [142] and alphaviruses [109] are the two pathogens identified to date that are known to induce a significant increase in the circulating plasma levels of sP-selectin. In particular, patients infected by the mosquito-borne alphavirus responsible for Chikungunya fever have strong platelet activation and increased levels of sP-selectin compared to healthy controls. Nonetheless, the in vitro infection of platelets from healthy donors with Chikungunya virus only partially reproduces the phenotype observed in patients, probably because in vivo infection with CHIKV can trigger systemic generation of additional stimuli that promote platelet activation and the shedding of P-selectin [109].

### 4.4. sGPIbα, sGPVI, and sCLEC2

GPIbα and GPVI are known to be shed by metalloproteases of the ADAM family [143]. 

The shedding of GPIbα is mediated by ADAM17 upon a GPIbα–VWF interaction and supports the release of the receptor ectodomain (sGPIbα’s molecular weight is unknown) [144,145]. Increased levels of circulating sGPIbα and VWF have been detected in malaria patients [146]. The functional consequences of GPIbα shedding in malaria infection are still unknown, but it has been suggested that it may represent a negative regulatory mechanism of the VWF-mediated cytoadherence of infected red blood cells to the activated endothelium [147].

Soluble GPVI (sGPVI, 55 kDa) is a useful biomarker of platelet activation and thrombotic risk and is released from the platelet surface by ADAM10-dependent proteolysis [148]. Elevated levels of sGPVI have been found in several disease conditions, but its involvement in infections remains poorly investigated. Circulating sGPVI concentrations have been found to be increased in the plasma of septic patients, probably by a mechanism dependent on platelet exposure to the fibrin. Interestingly, the plasma levels of sGPVI correlated with sepsis onset and patient mortality [149].

CLEC2, as described in the previous section, is a platelet receptor actively involved in the body’s response to viruses. The full-length form can be proteolytically cleaved (sCLEC-2, 25 kDa) upon platelet activation, and possible involvement of MMP-2 in this cleavage has been suggested. Despite the importance of CLEC-2 as PRRs, the role of its shedding in infection is still unknown. However, increased levels of sCLEC-2 have been observed in patients with thrombotic microangiopathy and acute coronary syndrome [150], suggesting that sCLEC-2 may serve as a marker of platelet hyperactivation in different contexts. Moreover, a recent study developed a specific index, the ratio of sCLEC-2 levels and platelet count, useful for following the progression of sepsis-induced coagulopathy (SID) in septic patients [151].

## 5. Conclusions

The evolution of our understanding of platelet function beyond haemostasis and thrombosis is continually expanding and has led to the discovery of several platelet roles in infectious diseases.

Many of the platelet responses to pathogen invasion require the interplay of haemostasis, inflammation, and immunity. The platelet–pathogen interaction leads to platelet activation, which in turn results in platelet aggregation, thrombosis, increased interactions with leukocytes, and cytokine release. Platelets are thus important coordinators of both inflammation and immunity, and most platelet responses are common to both frameworks. However, some platelet responses are specifically relevant for host defence (e.g., the trapping of pathogens), whereas others have pronounced inflammation-regulatory functions (e.g., the release of inflammatory molecules and chemotaxis). Unravelling the different specific pathways regulating platelet function in haemostasis, immunity, and inflammation is going to be a complex and stimulating challenge for the future. 

Despite the role of platelets in viral and bacterial infections having been thoroughly explored in the last few years, little is known about their role in parasitic infections. Therefore, a massive amount of work still needs to be conducted to completely reveal the actual potential of platelets in pathogen detection and eradication. It is expected that platelet membrane receptors and signalling pathways, with little recognized function in haemostasis and thrombosis, may provide critical features in the context of infection. Similarly, novel membrane proteins may be uncovered as platelet sensors of PAMPs and DAMPs, leading to the discovery of novel molecular mechanisms in immunity. A full understanding of the platelet involvement in the innate immune response may lead to the development of novel therapeutic approaches to fighting pathogens and limit potentially fatal consequences, such as infection-induced thrombosis.

## Figures and Tables

**Table 1:** Platelet-mediated responses in infection.

Direct Platelet-Mediated Responses	References
Immunothrombosis	[4,5]
Pathogen trapping	[6,7,8,9]
Release of antimicrobial effectors	[10,11,12]
Expression of antiviral molecule	[13]
**Indirect Platelet-Mediated Responses**	**References**
Neutrophil activation and NETosis	[14,15,16,17]
Shuttling of blood-borne bacteria to CD8α+ dendritic cells	[8]
CD4^+^ T-cell differentiation	[18,19]
Induction of Ig isotype switching	[20,21]
Release of pro-inflammatory molecules	See Table 2
Leukocyte recruitment	[8,10,14,22,23,24]

**Table 2:** Molecules released from platelets during infection.

Molecules	Effect
PF4	First-line defence against invading pathogens [28]. Intraerythrocytic parasite killing [11,29] and activation of neutrophils [14,15,16,17].
Soluble CD40 Ligand	APC maturation and activation, production of interferon-γ by T cells, and differentiation of naïve T cells into effector cells [30,31]. Stimulation of dendritic cells [32]. Regulation of B-cell isotype switching and CD8 T-cell responses.
TGF-β1	Conversion of conventional CD4+ T cells into induced regulatory T cells [33]
PDGF	Attraction of monocytes to the site of the vascular injury and production of superoxide anions from eosinophils [34].
VWF	Increase of inflammation and neutrophils extravasation [35,36,37].
SDF-1	Potent chemoattractant of monocytes, T and pre-B lymphocytes [38], and dendritic cells [39]. Effect on T-cell rolling and tight adhesion to activated endothelial cells [40].
ADP	Increase of antigen endocytosis and processing [41].
Serotonin	Stimulation of monocytes [42] and lymphocytes [43].
P-selectin	Recruitment and activation of both innate and adaptive immune responses.

PF4: Platelet Factor 4; TGF-β1: Transforming growth factor beta 1; PDGF: Platelet-derived growth factor; VWF: von Willebrand factor; SDF-1: Stromal cell-derived factor-1.

**Table 3:** Platelet receptors involved in pathogen recognition.

Receptor	Pathogens/PAMPs
***PRRs***	
CLRs	
- CLEC-2	HIV [66], DV [67], CpG ODN [68]
- DC-SIGN	HIV [66], DV [69]
TLRs	
- TLR2	Periodontopathogens [70], HCMV [58], Pam3CSK4 [71]
- TLR4	LPS [72,73,74]
- TLR9	CpG ODN [75]
NLRs	
- NLRP3	DV-induced ROS products [76]
- NOD2	MDP [77]
***Haemostatic Receptors***	
GPVI	HCV [78], SSL5 [79,80], CpG ODN [81]
GPIb	SSL5 [82], SrpA [83], GspB, Hsa [84]. Protein A (SpA) [85], H. Pylori [86]
Integrin αIIbβ3	Hantavirus [87], Adenovirus [88], SSL5 [82], SdrG [89], PadA [90], IsdB [91], FnBPA, FnBPB [92], ClfA, ClfB [93]
Integrin α2β1	Rotavirus [94]
FcγRIIA	IgG-opsonized cells [95], IAV (H5N1) [96], FnBPA, FnBPB [92]
P2Y12	CpG ODN [68]

ClfA and ClfB (clumping factor A and B of *Staphylococcus aureus*); CpG ODN (unmethylated cytosine-phosphate-guanine oligodeoxynucleotides); DV (dengue virus); FnBPA and FnBPB (fibronectin-binding proteins A and B of *Staphylococcus aureus*); GspB and Hsa (surface proteins of *Streptococcus gordonii*); HCMV (human cytomegalovirus); HCV (hepatitis C virus); HIV (human immunodeficiency virus); IAV H5N1 (avian influenza A virus of the H5N1); IsdB (iron-regulated surface determinant, IsdB, of *Staphylococcus aureus*); LPS (lipopolysaccharide); MDP (muramyl dipeptide); SSL5 (staphylococcal superantigen-like protein 5); PadA (platelet adherence protein A of *Streptococcus gordonii*); Pam3CSK4 (Pam3CysSerLys4); Protein A (Spa) (surface protein of *Staphylococcus aureus*); SdrG (*Staphylococcus epidermidis* serine–aspartate repeat protein G); SrpA (*Streptococcus sanguis* platelet adhesin called serine-rich protein A).

## Data Availability

Not applicable.
